# ANA positivity in juvenile idiopathic arthritis: make a difference in a Colombian children population?

**DOI:** 10.1186/1546-0096-12-S1-P205

**Published:** 2014-09-17

**Authors:** Clara Malagon, Angela C Mosquera, Camilo Vargas

**Affiliations:** 1 Cundinamarca, Universidad el Bosque, Bogota, Colombia

## Introduction

Juvenile idiopathic arthritis is the most common cause of chronic arthritis in children. JIA is a heterogeneous disease that has been classified by the International League of Associations for Rheumatology (ILAR) on seven different subtypes according to the most relevant clinical and serological features. Previous studies have demonstrated an association between the presence of antinuclear antibodies (ANA) and features like: early-onset oligoarticular disease, female predominance, asymmetric arthritis and higher frequency of uveitis. There are limited data on the characteristics clinical and analytical into different subtypes of JIA in Colombian children and their relationship with type of onset and clinical course.

## Objectives

To describe a group of Colombian patients with JIA and analyze the clinical features according to ANA positivity (more or equal to 1;160 was considered as positive).

## Methods

This is a descriptive case series study. Includes patients from three pediatric rheumatology clinics in Bogotá, Colombia. The associations between clinical and laboratory parameters were analyzed using chi square test. P values less than 0.05 were considered as significant.

## Results

A total of 308 patients were included. The distribution of JIA patients according to ILAR category were: systemic arthritis 23 (7.5%), oligoarticular persistent 86 (28%), oligoarticular extended 8 (2.5%), RF positive polyarthritis 21 (6.8%), RF negative polyarthritis 71 (23%), ERA 94 (30%), psoriatic arthritis 4 (1.3%) and undifferentiated 1 (0.3%). 64% were female and mean follow up time was 55 months (9-192). Uveitis was present in 19 (6.2%) patients, 68% had oligoarticular extended disease. Of the study cohort, 54 (17.5%) patients were reported as ANA positive (≥1:160). Represented by oligoarticular persistent, RF negative polyarthritis, RF positive polyarthritis and ERA, (46%, 22%, 13% and 13%), respectively. ANA positive group, 81% were female sex and mean age of arthritis onset was 7y (2-14y). ANA dilution was in 65% ≤1:320. Uveitis was more frequent in ANA positive patients (17% vs. 0.4% *p*=0.002), in oligoarthritis and in early-onset JIA <5y (*p*=0.003).

## Conclusion

ANA positive patient group had a higher rate of female sex, younger age of onset and higher rate of uveitis. These findings are consistent with those reported by other authors. Furthermore, this cohort differs from that reported in the literature for the high frequency of ERA subtype and 13% of ANA positivity in these group of patients.

## Disclosure of interest

None declared.

